# Optical singularity protractor for rotating metrology with neuromorphic sensing

**DOI:** 10.1038/s41377-026-02357-8

**Published:** 2026-07-14

**Authors:** Zhe Weng, Yiyu Zhao, Zhiming Qing, Zhi-Cheng Ren, Wenxiang Yan, Xi-Lin Wang, Jianping Ding, Hui-Tian Wang

**Affiliations:** 1 National Laboratory of Solid State Microstructures and School of Physics, Nanjing University, Nanjing, China; 2 Collaborative Innovation Center of Advanced Microstructures, Nanjing University, Nanjing, China; 3 Collaborative Innovation Center of Solid-State Lighting and Energy-Saving Electronics, Nanjing University, Nanjing, China; 4 Collaborative Innovation Center of Extreme Optics, Shanxi University, Taiyuan, China

**Keywords:** Optical physics, Optical techniques, Imaging and sensing

## Abstract

The development of singular optics and vortex beams has facilitated numerous novel optical applications, with optical sensing being one prominent direction. By leveraging the rotational energy flow properties of vortex beams, rotational Doppler effect-based sensing for rotating objects has achieved significant progress. However, due to fundamental constraints of orbital angular momentum (OAM) and interferometry-based experimental methods, rotational Doppler sensing typically requires stringent axial alignment (single-point or multi-point) in practical scenarios, which limits their applicability. To address these limitations, this study focuses on developing rotating metrology utilizing singularities inherently associated with vortex phases, akin to the recently developed singularity ruler employing intrinsic/extrinsic modification of OAM density. In this work, we propose a sensing technique termed the “Optical Singularity Protractor (OSinP)”, which utilizes the angular velocity of singularities to reflect the rotational speed of optical fields. To extend the stable singularity ruler to dynamic rotation sensing, we developed a real-time neuromorphic singularity perception system capable of acquiring microsecond-level-resolution time-series data regarding singularity positions and movement directions, thereby enabling the sensing of optical field rotation velocity. Furthermore, we challenged the OSinP system with practical constraints, notably off-axis rotation and non-steady-state velocity, to verify its robust operational capabilities. This singularity sensing technique distinguishes itself from traditional rotation sensing by introducing neuromorphic computing into topological structured light and holds promise for neuromorphic applications in fields with stringent energy-efficiency requirements, such as astronomical metrology and embodied intelligence.

## Abstract (web summary)

By integrating neuromorphic computing with topological structured light, this work propose a sensing technique that utilizes the angular velocity of singularities to directly reflect the rotation speed of optical field.

## Graphical abstract

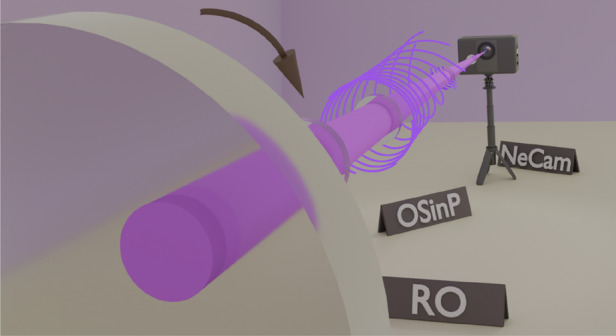

## Introduction

Singular optics^1^ primarily investigates structured light, especially vortex beams. Vortex beams exhibit a helical phase structure whose phase indeterminacy^2^ leads to optical singularities. Specifically, for a vortex phase exp(*imφ*) (with *m* being the topological charge representing quantized OAM and *φ* being the azimuthal angle), its phase gradient along the optical axis becomes infinite.^3^ This azimuthal phase gradient can form a high-dimensional, spatially infinite superposition state corresponding to the OAM spectrum of spatial eigenmodes,^4,5^ with the resulting state being referred to as a composite vortex beam. Beyond their distinctive phase structure and hollow intensity profile, vortex beams demonstrate unique energy flow characteristics manifested through the angular relationship *m*/*kr* between the Poynting vector and propagation direction (with *k* as the wave vector and *r* the radial coordinate).^6^ While numerous classical^7–9^ and quantum^10–12^ applications have been developed based on vortex beams’ topological charge and energy flow properties—spanning optical communication,^13,14^ manipulation,^15^ and metrology,^16,17^ relatively few studies have explored applications centered on the singularities themselves,^18–22^ which motivates our present work on optical sensing, especially rotational sensing, through exploiting fundamental singularity properties.

In optical sensing, the relative motion of optical fields has a well-established physical interpretation: the Doppler effect.^6,16^ Whereas the linear Doppler effect is used to measure translational velocity a target object relative to a detector, the rotational Doppler effect characterizes the object’s rotation by analyzing the field it emits.^23–25^ Studies have shown that the rotational Doppler shift magnitude is proportional to the topological charge of optical field, manifesting as time-varying phase,^26^ or frequency shifts in Fourier optics terminology. This enables OAM spectrum analysis for Doppler spectra.^27^ However, the extrinsic properties of OAM limit Doppler shift detection—different reference points selections lead to different OAM spectra, appearing as spectral broadening that causes Doppler spectrum aliasing and overlapping confusion in off-axis scenarios.^28^ Additionally, conventional image sensors have practical limitations in detecting phase,^29^ requiring interferometric measurements (typically divided into common-path or heterodyne detection) that introduce sign degeneracy (preventing redshift/blueshift discrimination). Compared to heterodyne detection, common-path interferometry can reduce the influence of linear Doppler effects and optical path noise, with composite vortex beams being the most common choice. For detection, Doppler shifts enable time-frequency spectrum measurements in the far field, typically using Fourier transforms of photodiode (PD) signals.^30^ Single-point PD detection requires no spatial resolution, but to overcome alignment issues and sign degeneracy, additional spatial information—such as multi-point detection^31^—is needed. In practice, off-axis misalignment frequently occurs at either the rotation axis or the far-field/conjugate detection axis, and although multi-point detection can mitigate this, it practically demands more stringent optical alignment of multiple detectors, thereby increasing experimental complexity. In summary, conventional PD-based methods acquire Doppler shifts through far-field detection without spatial resolution but require precise multi-point detection and alignment when addressing practical scenarios such as off-axis rotation and direction determination. In contrast, our approach measures the angular velocity of singularities through a unique surface-array gradient detection mechanism that relaxes these stringent requirements.

**Fig. 1:**
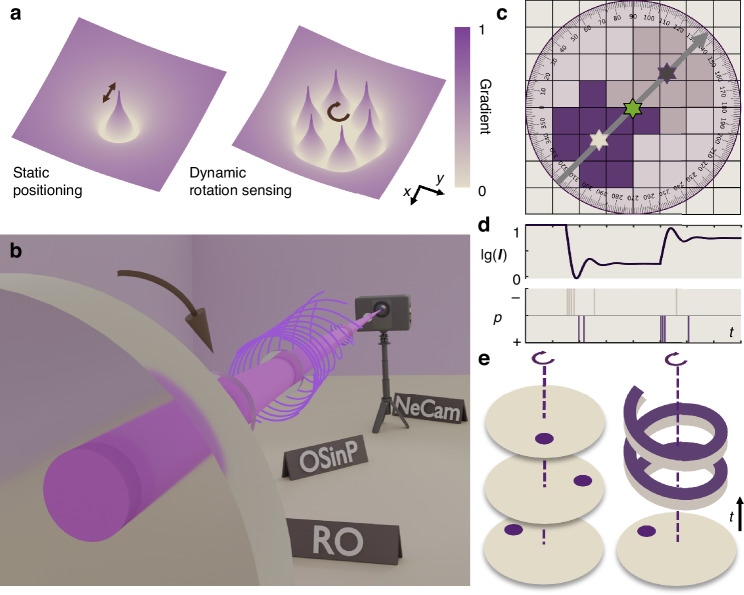
Principle and implementation of the optical singularity protractor (OSinP). **a** The sharp intensity gradient distributions of phase singularity: left panel for on-axis singularity, right panel for off-axis singularity group. Traditional static 3D measurement (left) utilizes displacement of singularities (double-headed arrow), whereas dynamic rotational velocity (right) detection considers rotation of singularity groups. **b** Foreshortened schematic of OSinP experimental setup: foreground RO (rotating object) and background NeCam (neuromorphic camera). Arrows indicate rotation direction of the rotating object (with a foreground frosted prism as example). Rotation velocity of the rotating object is measured contactlessly by recording singularity trajectories (purple winding traces) of structured laser beams (transmitted purple light) via the background NeCam. **c** Calculating rotation angle and angular velocity through array-based event response of singularities. Singularity movement generates events with positive (deep purple) and negative (gray) responses (details in **d**). Star markers indicate positive event centers (beige), negative event centers (dark gray), and total event centers (green). Singularity motion angle (gray arrow) and velocity are calculated based on these centers. **d** Event-response principle: logarithmic intensity-gradient thresholding in hardware. *lg*(***I***) shows a normalized logarithmic intensity time series, with *p* is the corresponding asynchronous event responses (trigger when gradient changes exceed threshold: positive for intensity increase, negative for decrease). **e** Low-frame-rate intensity capture (left panel) vs. high-sampling-rate event response (right panel) for dynamic rotating objects, using a rotating disk containing off-axis dark points (purple) as example

In this work, we focus on the characterizing role of singularities in rotational sensing, unlike traditional approaches utilizing the energy flow properties of vortex beams. As zero points in OAM density, singularities provide subwavelength-scale superoscillatory features that establish them as competitive sensing benchmarks. Recently, analogous to mass centroid and plumb lines in classic mechanics, singularities and OAM null lines have served as positioning rulers^21^ in three-dimensional static measurements. Here we extend this concept to dynamic rotating object sensing (Fig. 1a), establishing an analogy with the relationship between moment of inertia and angular momentum in center-of-mass reference frames, which we term the “optical singularity protractor (OSinP) ” (Fig. 1b). By measuring the angular velocity in singularity group of the optical field (Fig. 1c), OSinP determines the rotational speed of ROs (rotating objects). The primary challenge in dynamically characterizing singularities is addressed through an innovative neuromorphic camera (NeCam) approach that relies on gradient-based event detection rather than absolute intensities (Fig. 1d, e). Furthermore, we investigate OSinP’s performance in practical rotational scenarios including off-axis rotation and non-steady-state velocities with alternating directions, demonstrating OSinP’s robustness. Our work pioneers the application of event-based sensing via structured light fields, opening new avenues for further exploration in metrology with topological light.

## Results

### OAM, rotation and singularity

**Fig. 2:**
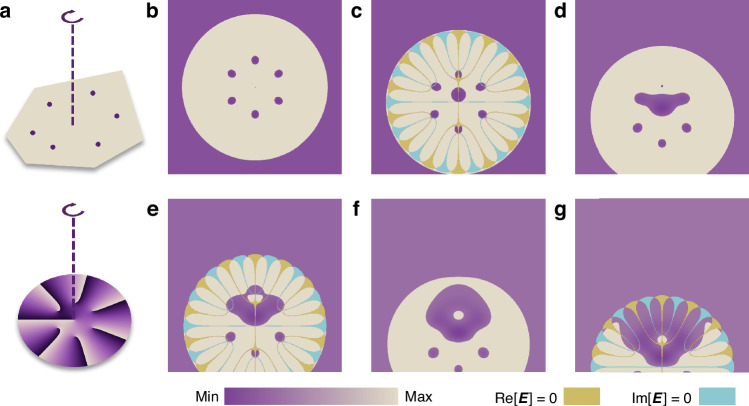
Rotation model and extrinsic characteristics of OAM density and its null lines. **a** Analogy in rotating systems: rigid-body rotation of centroid-of-mass group (purple points); optical-field singularity group. **b**–**g** OAM density distributions of a composite vortex beam (details in Fig. 3) with the image center as the reference point, showing how varying beam displacements in the transverse plane lead to distinct OAM density distributions (downward beam displacement is equivalent to upward shift of reference position). Displacements **b**–**g** correspond to 0, 0.675, 1.35, 1.875, 2.625, and 3.75 times the beam waist radius *w*_0_, respectively. Saturation of OAM density highlights the null lines at the interface between positive (beige) and negative (purple) regions. In **c**, **e** and **g**, field zeros of ***E*** are plotted on the corresponding OAM density maps: blue = imaginary part, brown = real part; intersections of both parts indicate phase singularities, which are traversed by OAM null lines as does the central reference point

The rotation of a rigid system or an optical field is intrinsically linked to its OAM density distribution (Fig. 2a). The OAM density of an optical field, ${\boldsymbol{J}}=({{\boldsymbol{r}}}_{o}+{\boldsymbol{r}})\times {\boldsymbol{p}}$, can be derived from the energy flow ${\boldsymbol{p}}\propto \frac{1}{2{\omega }_{0}}\mathrm{Im}[{{\boldsymbol{E}}}^{\ast }\times (\nabla \times {\boldsymbol{E}})]$, where ***E*** stands for the electric component of a monochromatic optical field with frequency *ω*_0_. For light carrying longitudinal OAM (rotation along *z*-axis), we primarily focus on its *z*-component *J*_*z*_. The OAM density consists of intrinsic and extrinsic components, which are distinguished by their dependence on the reference coordinate system. While the OAM density of light varies with the choice of reference point ***r***_*o*_, certain invariant features persist. Specifically, regardless of the reference point selection, the null lines of OAM density always pass through both the reference point and the singularities—a key property that underlies the development of singularity rulers^21^ for static field localization. Figure 2b–g reveals that, unlike the stable singularity lines, the OAM null lines in a singularity system exhibit more complex behavior. Off-axis singularities generate multiple OAM null lines instead of a single line passing through both the singularity and the reference point. These multiple null lines partition the OAM density into alternating positive and negative regions, which fuse or emerge depending on the reference point selection. Overall, irrespective of the reference point selection, all null lines must pass through the singularities, though not necessarily through the reference point (Fig. 2g).

We draw an analogy with the moment-of-inertia model of rigid bodies to demonstrate the unique properties of singularities in dynamic rotational speed sensing, similar to the plumb-line centroid model used in singularity rulers (Fig. 2a). In this framework, the correspondence between singularities and mass centroid is extended to singularity groups analogous to particle systems. As described by the Pappus centroid theorem^32^ or parallel axis theorem,^33^ the angular momentum *L*_*z*_ of a rigid body relates to the angular velocity *ω*_*i*_ of its mass centroids, where the choice of reference point affects the change of moment of inertia *I*_*i*_ + Δ*I*_*i*_ of mass centroids, consequently altering angular momentum density. The total angular momentum thus becomes ${L}_{oz}={\sum }_{i}({I}_{i}+\Delta {I}_{i}){\omega }_{i}$. This analogy motivates us to focus on the angular velocity of singularity groups within rotating optical fields. For rotational sensing, measuring the angular velocity of singularities in these groups enables extraction of the rotating object’s velocity.

**Fig. 3:**
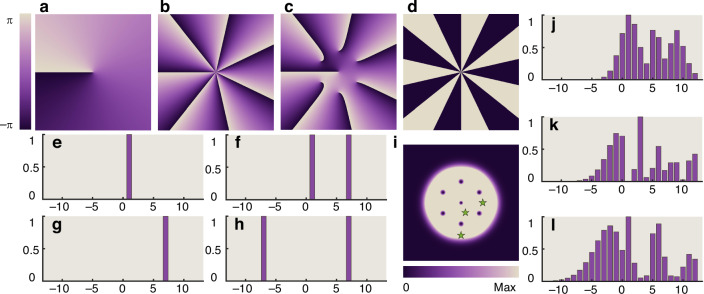
Composite vortex beams and their OAM spectra (LG modes: *p* = 0). **a**–**d** Phase profiles: LG beams carrying topological charge *m* = 1 (**a**) and *m* = 7 (**b**), and composite vortex beams (1,7) (**c**) and (-7,7) (**d**). **e**–**h** OAM spectra corresponding to **a**–**d** subpanels. **i** Saturated intensity profile of the (1, 7) off-axis singularity beam, indicating singularity positions. **j**–**l** Spectral broadening of OAM spectra under off-axis reference point displacement. Subpanels **j-l** correspond to displacements at the three reference points (asterisk markers in (**i**)), demonstrating increasing broadening with displacement from the beam center

For practical rotating optical fields, composite vortex beams—superpositions of vortex modes (e.g., $E(r,\varphi )={\sum }_{m}{c}_{m}{E}_{m}(r)\exp (im\varphi )$, *c*_*m*_ are mode coefficient of vortex modes)—are typically employed in measurements. A canonical example is the symmetric superposition of ±*m* Laguerre-Gauss (LG) beams ($L{G}_{p}^{m}(r,\varphi )$) with identical radial index *p* and opposite azimuthal index *m*,^34^ with phase pattern shown in Fig. 3d and mode spectrum in Fig. 3h, denoted as (-7,7) for *m* = 7. We use LG modes with *p* = 0 and different *m*, such as *m* = 1 (Fig. 3a, e) and *m* = 7 (Fig. 3b, g). For rotational sensing compatibility, we utilize (1,7) composite vortex beams but focus on singularity behavior (see Supplementary Note 1). As shown in Fig. 3c, f and i, the (1,7) composite vortex beam generates a singularity group of six off-axis singularities and an on-axis singularity, each carrying unit topological charge ( | *m* | = 1). Under this illumination condition, the rotational Doppler shift is Δ*f* = 6*Ω*/2π (For detailed calculations, refer to Supplementary Note 1). These off-axis singularities—identified by either zero-intensity points or maximum-intensity-gradient locations—exhibit a rotational speed of *Ω*. This characteristic remains invariant under off-axis misalignment. The on-axis singularity is an exception: while it exhibits zero linear velocity under coaxial conditions (as it lies on the rotation axis), it acquires a non-zero rotational speed in the misaligned case. Figure 3j–l demonstrates progressively broadened OAM spectra with increasing off-axis distance, leading to Doppler spectral overlap. Notably, singularities maintain a fixed rotational speed *Ω*, while their linear velocities scale with off-axis displacement. This characteristic holds generally for the singularity behavior in any composite vortex beam formed by two positive modes, thereby establishing a singularity-based method to characterize optical field rotation.

### Rotation velocity detection via OSinP

Building upon the established relationship between singularity velocity and optical Doppler effects, the key remaining challenge is how to efficiently acquire singularity speeds in a fast, lightweight, and widely applicable manner. Our solution leverages gradient sensing: singularities, as abrupt transition points in optical fields, are more effectively resolved through spatial gradient detection **Δ*I*** than temporal integration ***I***. Fortunately, NeCam, a novel gradient sensor that operates asynchronously, has been developed.^35–38^ In this architecture, each pixel in NeCam detects logarithmic intensity gradient changes $|\Delta \,\mathrm{lg}({\boldsymbol{I}})|\ge C$ through adjustable thresholds (*C*) and returns asynchronous data indicating the direction of change (Fig. 1d). This neuromorphic mechanism provides high information throughput, wide dynamic range, and other advantages that perfectly meet singularity detection requirements. For example, the sensor’s event-driven operation enables real-time tracking of singularity motion without conventional frame-based system latency, as shown in Fig. 1e. By directly capturing intensity gradients at microsecond timescales, OSinP offers a more efficient solution for dynamic singularity characterization in practical rotational sensing applications. A comparison of the singularity precision achieved by our method and conventional intensity-based methods is provided in Supplementary Note 2.

**Fig. 4:**
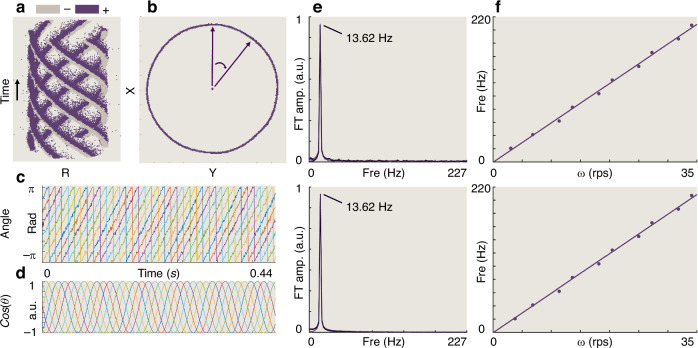
Experimental validation of two singularity-based rotation speed sensing methods. **a** Time-series polarity data of rotating singularities. R represents the (x, y) plane. **b** XY distribution of singularity positions with calculated rotation center. **c**, **d** Angle extraction for six off-axis singularities (clockwise defined as positive): **c** Time-series of angle derived from polarity event; **d** Time-series of cosine angles between singularity positions and geometric center. The individually calculated values for six singularities are represented by six distinct colors. **e** Fourier spectra calculated from both time-series in **c** and **d** (top: polarity-derived angles in **c**; bottom: position cosine angles in **d**; same for **f**). **f** Linear relationship between measured rotation speed (*x* axis) and preset Doppler frequency shifts (*y* axis)

We first describe how to stably obtain the velocity and rotation direction of singularities in OSinP. Figure 4a displays a raw echo signal $[r(x,y),p=\pm 1,t]$ with the optical field rotation speed set at 82.86 rps. The positive and negative polarity events *p* represent positional changes of six off-axis singularities, exhibiting helical patterns along the time axis from 54.153 *ms* to 104.137 *ms*, where the polarity data indicates the direction of singularity linear speed vectors. To extract the speed information, we perform frame compression on event data using a 660 *μs* window, representing the actual temporal resolution 1515 Hz. Using Density-Based Spatial Clustering of Applications with Noise (DBSCAN) algorithm,^39^ we utilized unsupervised clustering of the events in each frame to determine each singularity position ${\vec{s}}_{i}(x,y,t)$ and centers of opposite polarity events ${\vec{p}}_{i\pm }(x,y,t)$, then calculate singularity speed vector directions ${\theta }_{i}(t)=\arccos [{\vec{p}}_{i+}\cdot {\vec{p}}_{i-}/|{\vec{p}}_{i+}||{\vec{p}}_{i-}|]$, with results shown in Fig. 4b, c. Circular trajectories of the six off-axis singularities are revealed in Fig. 4b, where their calculated geometric center ${\vec{s}}_{0}(x,y)$ serves as the rotation center. Unlike conventional rotation sensing, OSinP requires no strict axial alignment—only encompassing the singularities movement—to determine the rotation center from measured data. In experiments, the incident beam radius was 0.836 *mm* (imaging plane after *f* = 100 *mm* zoom lens), while the NeCam (Sony IMX636ES in this work, 1/2.5”, 6.22×3.50 *mm*) provided generous alignment tolerance compared to PD-based methods.

Based on the positions and speed directions of singularities, we propose two methods to calculate the rotational frequency shift. The speed-vector method utilizes ${\theta }_{i}(t)$ (Fig. 4c), while the center-cosine method utilizes ${\vec{s}}_{i}(t)$ to compute the cosine angle $\cos \,{\theta}^{\prime}_{i}(t)={\vec{s}}_{i}\cdot {\vec{s}}_{0}/|{\vec{s}}_{i}||{\vec{s}}_{0}|$ between the singularity’s motion trajectory and the geometric center ${\vec{s}}_{0}(x,y)$ (Fig. 4d). Both yield the angular velocity of singularities. Defining clockwise rotation as positive, the event data in Fig. 4 reveals the positive rotation direction (Fig. 4b–d). For constant-speed rotation, the rotational frequency shift can be extracted through Fast Fourier Transform (FFT). Figure 4e demonstrates a measured rotational frequency shift of 13.62 Hz (relative error 1.37%) that is obtained from the FFT spectrum peak, with all singularity time series showing identical results. Notably, the FFT spectrum in Fig. 4e exhibits exceptional purity compared to the significant background interference typically observed in PD-based measurements. This superiority stems from the intrinsic alignment-free nature of OSinP, which effectively eliminates higher-order harmonics, combined with the data sparsity characteristic of the event-driven detection mechanism.

To evaluate OSinP’s universality and limits, we tested Doppler shift ranging from 0 to 220 Hz, with measurement results shown in Fig. 4f. The fitted measurements exhibit a linear growth relationship with the preset Doppler shifts, fully consistent with theoretical predictions. Both methods demonstrate nearly identical results, with deviations only occurring at low speeds (first data point). This discrepancy arises from reduced event responses induced by singularities at low rotation rates, affecting clustering algorithm stability (detailed analysis in Fig. 6). Additionally, as shown in Fig. 4c, e, using singularity speed vectors ${\theta }_{i}(t)$ introduces higher-frequency noise compared to trajectory-based ${\vec{s}}_{i}(t)$ methods, likely caused by trailing artifacts^40,41^ of NeCam (see Supplementary Note 3) and subsequent clustering errors, though without impacting frequency shift calculation. A quantitative estimate of the noise tolerance is provided in Supplementary Note 3. All Fig. 4 results were obtained under identical experimental parameters. Adjusting background intensity and hardware thresholds (*C*: contrast, bias, cutoff frequency, etc.) can enhance singularity performance for different rotation velocity (the parameters used in this work refer to Supplementary Note 3). Further improvements may incorporate advanced deep learning or spiking neural networks into OSinP.

### Multi scene detection: off-axis and non-stationary velocity

**Fig. 5:**
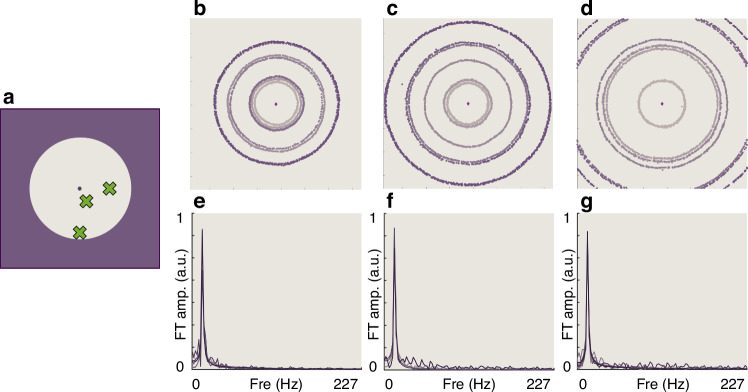
Off-axis rotation measurement results. **a** Schematic diagram of rotation centers (cross marks), using the same reference points as Fig. 3i. **b**–**d** XY distributions of singularity positions with progressive center-offset: **b**–**d** Rotation centers (cross in **a**) moving incrementally away from the beam center. **e–g** Fourier spectra calculated from the time-series data in **b**–**d**

To demonstrate OSinP’s practical utility, we examined two challenging scenarios: off-axis rotation and non-steady-state speeds with obtainable directionality. Off-axis rotation induces OAM spectral broadening, causing Doppler shift dispersion, while OSinP remains unaffected. Non-steady-state speeds showcases OSinP’s capability to track velocity variations and determine directionality. For off-axis rotation, we tested three distinct rotation axes (Fig. 5a) at identical rotational speeds, corresponding to the reference point selection in Fig. 3i. Figure 5b–d displays singularities identification results as the rotation axis progressively shifts from the center. The misalignment between rotation and optical axis generates seven concentric circles, comprising both moving off-axis singularities and an on-axis singularity. Crucially, the rotation center is directly computable from raw data without prior knowledge of axis position. FFT analysis extracts a consistent 13.62 Hz peak from all concentric trajectories (Figs. 5e–g), confirming rotational velocity at 13.62 rps. Compared to PD intensity measurements, singularity-derived velocity provides more direct frequency shift determination through spatial-domain analysis, circumventing OAM mode projection and its intrinsic spectral limitations.

**Fig. 6:**
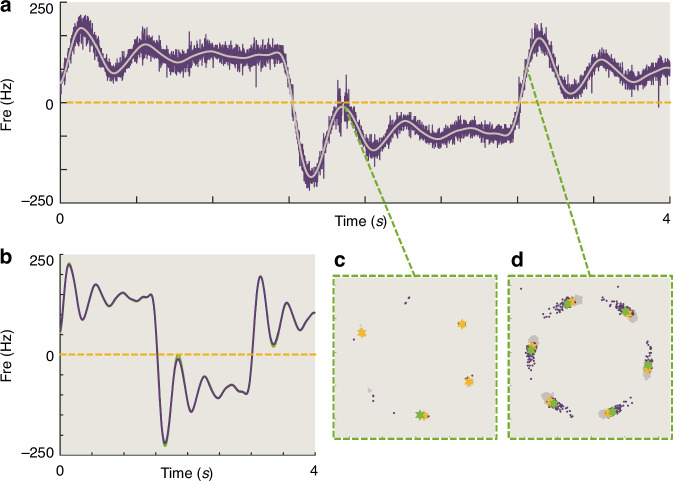
PID-type rotational speed measurement results. **a** Raw rotational velocity data (purple) vs. its FFT-filtered extraction (ivory). **b** Comparison between averaged multi-singularity results (purple) and preset speeds (green), with yellow dashed lines indicating zero-speed boundaries (clockwise above, counterclockwise below; also applies to the yellow line in **a**).**c**, **d** Event responses near zero-speed (**c**) vs. at positive speeds (**d**): Yellow stars mark current-frame singularity positions; green stars show previous-frame positions. Frames are extracted at positions indicated by green dashed lines in **a**

To rigorously evaluate the practicality of OSinP in dynamic scenarios, we implemented a nonlinearly varying velocity profile simulating proportional-integral-derivative (PID) control system^42^ with directional reversals (Fig. 6). Such nonlinear control systems are common in real-world rotational applications,^43^ with governing equations derived from damped systems. OSinP successfully characterized the velocity series (Fig. 6a) generated under a preset PID regime (*P* = 5.5, *I* = 270, *D* = 0.055) with preset Doppler frequency shifts of 140.5 Hz (0-1.5 *s*), -85.5 Hz (1.5-3 *s*), and 85.5 Hz (3-4 *s*), which resulted in the nonlinear responses documented in Fig. 6b. The raw time-varying velocity for each singularity was calculated directly from the difference in the received angle data. For acquired singularity velocity (frequency shift) data (Fig. 6a), we applied direct FFT-based low-pass filtering and averaged all off-axis singularities to eliminate localization errors. Specifically, a cutoff frequency of 15 Hz was applied to the Fourier spectrum of the purple time varying velocity signal in Fig. 6a to suppress high-frequency noise, resulting in the filtered signal shown in ivory. Physically, this cutoff frequency corresponds to the upper limit of detectable acceleration for non-stationary motion under the current experimental configuration, as determined by the preset PID parameters. The averaged multi-singularity velocity time-series (Fig. 6b) showed error accumulation near zero-speed transitions, inherent to the detection mechanism. As illustrated in Fig. 6c, d, near-zero velocities result in weak singularity responses, leading clustering and averaging inaccuracies that affect direction and velocity estimation. Integrating frame-based sensing could effectively mitigate these errors, particularly in scenarios with gradual speed variations.

## Discussion

It is important to emphasize that, although we compared the relationship between OAM density at mass centroids of particle systems and singularity group in optical field, these entities are not equivalent, as they fundamentally originate from distinct governing equations.^44^ In fact, the OAM density of optical fields more closely resembles the behavior of fluid flow^20^ than that of rigid bodies. Nevertheless, in the context of angular momentum exchange in light-matter interactions, singularities and centroids exhibit certain conceptual parallels. Our findings show that the location of singularities in an optical field can reflect its rotational velocity, typically arising from light-matter interaction—consistent with the theoretical framework of the rotational Doppler effect. Under such circumstances, extending OSinP to more practical scenarios necessitate consideration of various rotating media, with particular attention to media-dominated singularity phenomena. For instance, thin linear samples like rough surfaces, or scattering media that introduce perturbations in optical paths can lead to phase distortions within the singularities signal. Such effects could cause jitter in the singularities group or the detection of non-priori singularities. Fortunately, similar noise characteristics have been effectively addressed by the singularity clustering algorithm and signal averaging strategy of OSinP.

OSinP prioritizes rapid singularity measurements over single-frame localization accuracy, thereby representing a trade-off in measurement emphasis. Singularity tracking has long been a key focus in optical measurement, with various detection approaches having been developed, including near-field probes,^45^ fluorescence microscopy,^46^ and intensity saturation.^47^ Notably, OSinP leverages the NeCam’s intrinsic nonlinear intensity detection mechanism, which is functionally analogous to intensity saturation, to enable high-speed singularity tracking in metrology. In conventional optical metrology, sensing, and imaging, singularity-dominated phenomena such as speckle and turbulence often pose significant challenges, necessitating singularity tracking to locate singularities and mitigate their impact on measurement accuracy. We believe that OSinP’s capability for dynamic singularity tracking will prove beneficial in turbulent environments such as atmospheric or underwater settings—potentially through integration with advanced methods like Doppler effect tailoring^48^ or adaptive optics. In parallel, while recent advances have established singularities as metrological benchmarks, existing studies remain largely confined to static scenarios. Unlike “singularity ruler” approach, which sacrifices temporal resolution for spatial precision, OSinP leverages the redundant intensity resolution of zero-intensity singularities to enhance information capacity. We emphasize temporal resolution performance in singularity localization—a necessary compromise under data throughput limitations. Importantly, OSinP’s logarithmic advantage over traditional intensity-based detection is also manifested in singularity localization. By reducing excessive data resolution related to singularity position alone, we effectively expand the trade-off boundary in dynamic singularity tracking. Furthermore, regarding temporal limits, NeCam provides a temporal resolution of 1 *μs*—comparable to the bandwidth of commercial photodetectors (e.g., PDA50B2, Thorlabs) —which is sufficient for many rotational sensing scenarios.

We have developed two methods for estimating the angular velocity of singularities, each based on different singularity characteristics. The center-cosine method relies on spatial coordinates of singularities, with its accuracy depending on the sampling rate and precision of the rotation radius (note: the pixel size of NeCam used in our work is 4.86 *μm*). The speed-vector method derives from the asynchronous response amplitudes of singularity motion, which are influenced by background contrast and trailing artifacts (see Supplementary Note 3). Both methods highlight the spatial sparsity of the novel asynchronous response. Depending on rotation scenarios, the methods can be flexibly combined—for example, in off-axis rotations with large singularity radii or composite rotational velocities (Figs. 5 and 6). However, as event responses are triggered only by motion, measurements of static or quasi-static targets may exhibit errors (Fig. 6c, d). Possible solutions include introducing active detector motion^49^ or combining with frame-based sensors for joint measurements.^50^ Since optical singularities are characterized by sub-wavelength gradient features, their detection is inherently a sub-wavelength process. The resolution limit presented by the NeCam’s sampling rate can therefore be addressed by integrating a magnification system to match these scales, improving performance in applications like resolving closely spaced singularities.

We identify several avenues for extending OSinP’s experimental configuration. Given that rotational Doppler shifts are achromatic, and our experiments employed a wavelength-independent digital micromirror device (DMD) without phase modulation, OSinP is potentially applicable to broadband light sources. Moreover, owing to NeCam’s unique gradient-based response mechanism, OSinP is expected to exhibit robustness against background illumination (typically broadband white light). Finally, the moving singularities identified in this work arise from vortex beams composed of superposed eigen-LG modes—structures that are also feasible in fiber optics.^30^ Therefore, integrating OSinP with compact fiber-optic transceiver systems represents a promising future direction.

In summary, we have developed an optical singularity protractor (OSinP) that utilizes singularity tracking to characterize the rotational Doppler shift, enabling the sensing of optical field rotation speeds in various application scenarios. Unlike traditional far-field rotational Doppler sensing relying on OAM orthogonal spectrum or OAM density analysis, our approach demonstrates that the movement velocity of singularities in composite vortex beams—serving as zero points in OAM density—can equivalently represent rotational speeds of optical fields, analogous to the moment-of-inertia modelling in rigid body dynamics. Experimentally, we achieved real-time tracking of singularity positions and velocity vectors through relative gradient measurements using a neuromorphic sensor array, offering advantages such as alignment-free operation and high data efficiency. Based on OSinP framework, we proposed two methods to determine rotational frequency shift and direction by analyzing time series of singularity positions and velocity vectors along with their corresponding Fourier spectra. We validated OSinP’s performance in practical scenarios, including off-axis rotation and nonlinearly varying rotational speeds. Our work integrates a neuromorphic nonlinear sensing mechanism with the singular nature of topological structured light, extending the application scope of singularity-based metrology in light-matter interactions to the temporal domain. These findings could have potential for broader applications in next-generation computing and signal processing systems, including optoelectronic hybrid computing and neuromorphic intelligent sensing.

## Materials and methods

It is reasonable to use a controllable device to simulate physical objects for probing the principles of both static singularity metrology and rotational Doppler effects. The key consideration lies in the matching of temporal resolution between the object-simulation device and the detector, as well as the clarification of applicable scenarios. In our proof-of-concept experiments, a DMD (DLP9000X, Texas Instruments) was used to generate singularity beams and simulate rotating objects.^51,52^ Singularities group are generated through the holographic modulation of the incident laser beam. Simultaneously, the frequency shift is achieved via high-speed switching of the DMD in the form of global time-varying phase modulation. This corresponds to a scenario involving rotating transmissive objects, such as a rotating Dove prism or frosted prism. The DMD simulates only the pure Doppler frequency component of the light beam passing through the rotating objects, while multi-layer scattering effects from physical objects are not considered in this model. Compared to real objects, tunable DMD devices offer superior signal purity and adjustability, overcoming certain experimental limitations such as mechanical imperfections. A 532 *nm* Gaussian laser was incident on the DMD, undergoing complex amplitude modulation via superpixel hologram encoding.^53^ Composite vortex beams containing off-axis singularities were obtained through +1st-order filtering in a 4 f system. The experimental setup is shown in Supplementary Note 4. To simulate rotation, we leveraged the DMD’s high-speed refresh capability with onboard memory to load time-varying phase pattern corresponding to rotating object dynamics, allowing accurate comparison between preset and measured values—especially crucial for non-steady-state velocities. Due to the need for rapid switching across numerous holograms that generate rotational sequences, we employed Look-Up Table (LUT) mode to facilitate hologram transitions. In conventional rotational Doppler simulation, the motion of a rotating object at each moment is pre-recorded as holograms and sequentially stored in the DMD’s memory. This approach requires extensive sequential hologram transfers, and the simulation duration is limited by both the DMD’s memory capacity and the hologram transfer speed. However, we noticed that phase variations are wrapped, meaning that a complete rotation simulation only requires a specific set of holograms to form a LUT. While the DMD’s refresh time is shorter than NeCam’s frame compression duration, LUT storage of time-dependent phase profiles for a full rotation allowed extended simulations (e.g., 4 *s* as shown in Fig. 6) at microsecond-level updates. The resulting optical field was described by:

1 $$T={A}_{1}^{2}+{A}_{7}^{2}+2{A}_{1}{A}_{7}\,\cos \left(({m}_{7}-{m}_{1})\varphi +\frac{[n(t)/{N}_{L}]}{{N}_{L}}2\pi \right)$$

where *A*_*i*_ (*i* = 1,2,…,7) denotes the amplitude of corresponding LG modes (see Supplementary Note 1), $[\cdot ]$ represents modulo operation, *N*_*L*_ is the total number of LUT entries stored in the DMD’s memory, and *n*(*t*) is the current hologram index input as 9-bit binary data, which is the only data that needs to be transfer between the DMD and the PC. Compared to sequential hologram transfer, this LUT-based scheme effectively circumvents data transfer rate bottlenecks between DMD and PC, enabling extended and precise simulation of rotating fields using commercially available hardware.

## Supplementary information

Supplementary Information for Optical Singularity Protractor for Rotating Metrology with Neuromorphic Sensing

## Data Availability

All data that support the findings of this study are available within the article and supplemental document, or available from the corresponding author upon reasonable request.
