# Supplementary material for: Optical singularity protractor for rotating metrology with neuromorphic sensing
Source: Light Sci Appl. 2026 Jul 14;15:316. doi: 10.1038/s41377-026-02357-8 (PMC13369470; doi:10.1038/s41377-026-02357-8)
Supplement: Supplementary file 1 — Supplementary Information for Optical Singularity Protractor for Rotating Metrology with Neuromorphic Sensing [file 41377_2026_2357_MOESM1_ESM.docx]

**Supplementary Information for**

**Optical Singularity Protractor for Rotating Metrology with Neuromorphic Sensing**

Zhe Weng,^1, 2^ Yiyu Zhao,^1, 2^ Zhiming Qing,^1, 2^ Zhi-Cheng Ren,^1, 2^ Wenxiang Yan,^1, 2^ Xi-Lin Wang,^1, 2^ Jianping Ding^1, 2, 3, *^ and Hui-Tian Wang^1, 2, 4*^

^1^ National Laboratory of Solid State Microstructures and School of Physics, Nanjing University, Nanjing 210093, China.

### ^2^ Collaborative Innovation Center of Advanced Microstructures, Nanjing University, Nanjing 210093, China.

### ^3^ Collaborative Innovation Center of Solid-State Lighting and Energy-Saving Electronics, Nanjing University, Nanjing 210093, China.

### ^4^Collaborative Innovation Center of Extreme Optics, Shanxi University, Taiyuan 030006, China.

### * Corresponding author: jpding@nju.edu.cn; htwang@nju.edu.cn

### **Supplementary Note:**

### **1. Rotation of structured light and the Doppler effect;**

### **2. Singularity resolution with NeCam;**

### **3. Trailing artifacts of NeCam;**

### **4. Detailed experimental setup schematic of OSinP;**

### **Supplementary Reference.**

**Supplementary Note 1 – Rotation of structured light and the Doppler effect.**

For an optical vortex beam carrying topological charge *m*, its expression is given by:

(S1)

where *A*(*r*) represents the amplitude with azimuthal symmetry, $k=2\pi\omega_{0}/c$ denotes the wave vector, and $\omega_{0}$ is the angular frequency with *c* being the speed of light. When the beam undergoes rotation $\Omega$ and translation *v* relative to the detector, the received signal becomes^1-3^:

(S2)

This indicates that the signal is modulated by a time-varying phase, introducing additional optical path differences. The frequency shift manifests as phase variations, requiring interferometric detection with a reference plane wave *A*_ref_ = exp(*ikct*). The interference intensity is:

(S3)

For simplicity, radial modulation terms are omitted here. In practice, this interference occurs predominantly on the vortex ring *r*_0_ where *A*(*r*_0_) = *A*_ref_ maximizes the interference contrast. The beat frequency extracted at this radius *r*_0_ gives the Doppler shift Δ*f*:

$\Delta f=\frac{m\Omega+2kv}{2\pi}$ (S4)

This shift combines rotational and linear Doppler effects in a heterodyne configuration. For common-path interference using composite vortex beams with topological charges *m*_1_ and *m*_2_:

(S5)

yielding a pure rotational Doppler shift:

$\Delta f=\frac{(m_{1}-m_{2})\Omega}{2\pi}$ (S6)

Compared to heterodyne interference, the use of composite vortex beams offers the advantage of canceling out the linear Doppler effect, such that the interference frequency shift arises purely from the rotational Doppler effect. As a special case, when the composite vortex beam consists of two beams with opposite topological charges *m*, the resulting Doppler shift becomes $\Delta f=2m\Omega/2\pi$.

While Eq. (S5) neglects radial dependence, we now derive the influence of radial variation *A*(*r*) on the interference intensity. The intensity distribution serves as the coefficient for both direct current and alternating current terms, affecting the interference contrast $2A_{1}A_{2}/(A_{1}^{2}+A_{2}^{2})$. Taking LG beams as an example^4^, the amplitude of a vortex beam carrying only topological charge *m* (with mode index *p =* 0) at *z =* 0 is given by:

$A_{m}(r)=\sqrt{\frac{2}{\pi|m|!}}\left[\frac{\sqrt{2}r}{w_{0}}\right]^{|m|}\exp\left(-\frac{r^{2}}{w_{0}^{2}}\right)$ (S7)

where *w*_0_ is the waist radius of the light beam. Substituting into the contrast expression yields:

$\frac{2A_{1}A_{2}}{A_{1}^{2}+A_{2}^{2}}=\frac{ 2 \sqrt{\frac{|m_{1}|!}{|m_{2}|!}}\left[\frac{\sqrt{2}r}{w_{0}}\right]^{|m_{2}|-|m_{1}|} }{1+\frac{|m_{1}|!}{|m_{2}|!}\left[\frac{\sqrt{2}r}{w_{0}}\right]^{2(|m_{2}|-|m_{1}|)}}$ (S8)

This function reaches maximum contrast $\frac{2A_{1}A_{2}}{A_{1}^{2}+A_{2}^{2}}=1$ at $r\geq0$ when:

$r_{0}=\frac{1}{\sqrt{2}}(\sqrt{\frac{|m_{2}|!}{|m_{1}|!}})^{1/(|m_{2}|-|m_{1}|)}w_{0}$ (S9)

For composite vortex beams with positive *m*_1_ and *m*_2_, perfect interference occurs precisely at this radius, which coincides with the position of off-axis singularities. Moreover, the cleanest frequency spectrum for rotational Doppler shift extraction is obtained at this singularity ring. Fig. S1 presents the interference contrast for different topological charges. Beyond the peak value, the width of the contrast peak also plays a role, reflecting the intensity gradient near the singularities. In our experiments, we selected *m*_1_ = 1 and *m*_2_ = 7 to generate composite vortex beams, achieving optimal singularity centroid formation. Furthermore, by engineering perfect vortex beams through *A*(*r*) modulation, sharper singularity centroids can be obtained without altering their vortex characteristics.


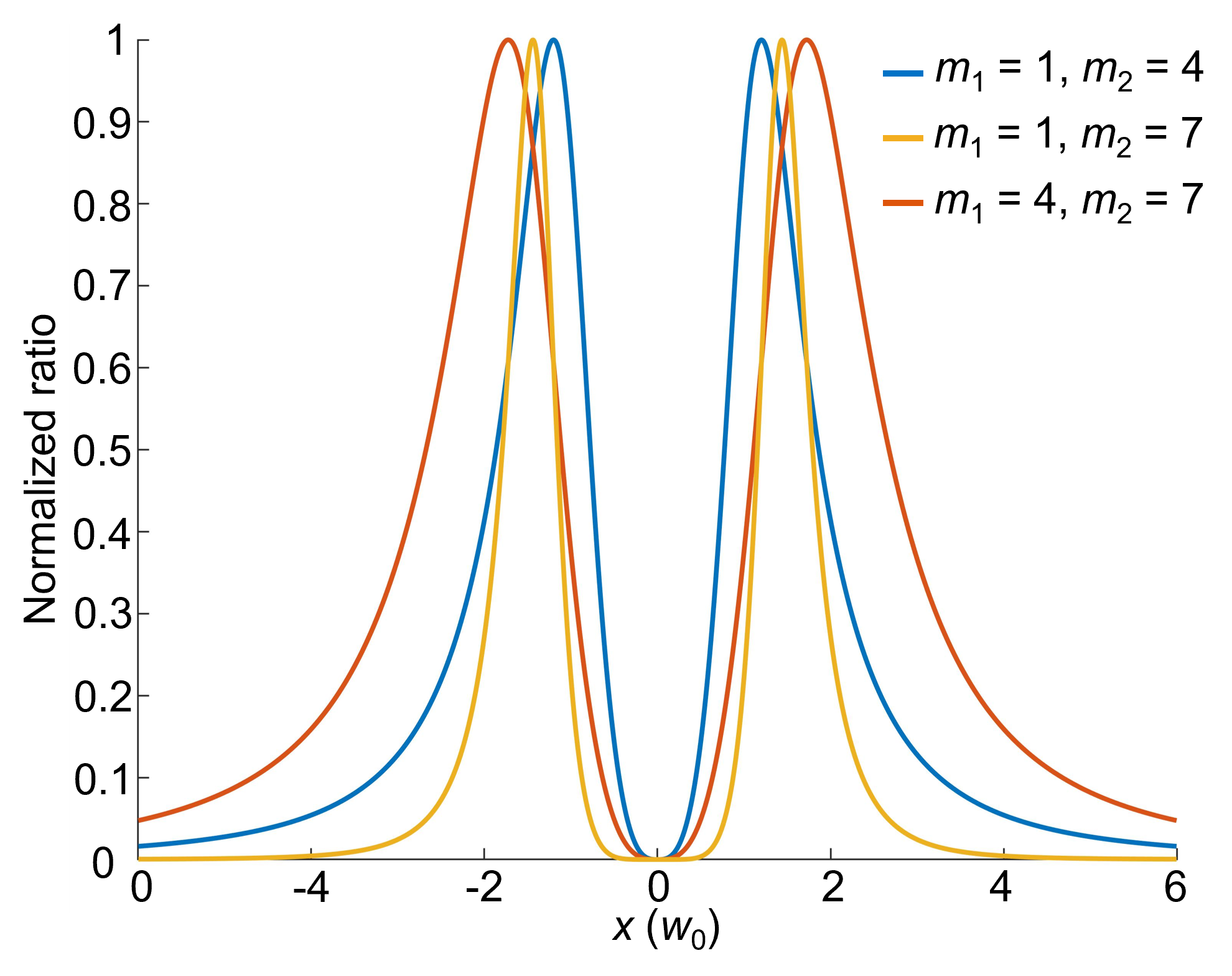


**Fig. S1:** **Interference contrast of composite vortex beams carrying different topological charges.** The *x*-axis in units of *w*_0_ and the *y*-axis showing normalized ratio.

In summary, the off-axis phase singularities utilized in this work originate from the perfect interference between distinct topological vortex rings. This interference process simultaneously bestows a corresponding Doppler shift upon phase singularities, resulting from the frequency difference between the constituent beams. From this perspective, interference is not only the reason for the existence of the phase singularities but also the very source of their associated Doppler shift. Furthermore, interference brings another critical attribute to phase singularities: a significant intensity gradient. This gradient is crucial for the efficiency of event-based sensing. For instance, by adjusting the threshold of NeCam, we can effectively distinguish the steeper gradient of phase singularities from the flat gradient of amplitude minima appearing at the beam edge, as shown in Fig. 1a and Fig. 3i. The physical property of phase singularities—that they possess a sub-wavelength-scale intensity gradient—is a primary reason for their role as a protractor.

**Supplementary Note 2 – Singularity resolution with NeCam.**

In this section, we employ a relative model to evaluate the singularity resolution of the NeCam and compare it with the conventional intensity saturation method. We characterize the localization resolution using the singularity of first-order vortex beam without loss of generality (Fig. S2a), given that the protractor’s singularity group also consists of first-order vortex. The NeCam detects logarithmic intensity gradient changes $|\Delta\lg(I)|\geq C$ through adjustable thresholds (*C*). To facilitate a comparison between conventional cameras and NeCam, we define a similar logarithmic response threshold for intensity-based method:

$C_{\text{ab}}=DR+\lg(E_{\text{gain}})+\lg(t_{\text{over}}/t_{\text{shutter}})$ (S10)

Here, *E*_gain_ ​represents the electronic gain, *t*_shutter_​ the shutter time, *t*_over_​ the overexposure time, and *DR* the dynamic range. We consider a typical camera that has a dynamic range of 80 *dB*, a shutter time *t*_shutter_​ = 10 *μs*, a full-well capacity of 45 *ke*⁻, and a quantum efficiency of 75%. Furthermore, the absolute light intensity should be specified for the intensity-based response. The simulated laser intensity is set to 6.5 *µW*, with a beam radius of 1.3 *mm*. Together, these intensity and responsivity parameters determine the position of the normalized intensity (where lg(***I***) = 0), thereby defining the camera's response radius for the singularity cluster (i.e., pixels with lg(***I***) < 0). The result, shown in Fig. S2b, indicates that under the given parameters, the camera fails to respond to the light intensity within 13 pixels around the singularity, corresponding to a localization limit of 13 pixels. The intensity saturation method improves this limit by extending *t*_over_​ or increasing *E*_gain_​, to provides additional Δ*C*_ab_​. However, this comes at the cost of an exponential increase in overexposure time, which is often impractical for dynamic measurements.

The singularity localization task is considerably effective with the NeCam. First, the asynchronous data generated by the intensity gradient provides additional information to assist in singularity localization, dividing the response of singularity into two half-regions corresponding to positive and negative events. As shown in Fig. S2c, the cluster radius decreases as the threshold selected for the NeCam increases. This is primarily due to the sub-wavelength gradient characteristic of singularities, where the theoretical gradient near a singularity should be infinite. However, in OSinP, rather than maximizing singularity resolution, it is often beneficial to appropriately broaden the singularity clusters to ensure a sufficient signal-to-noise ratio for the clustering algorithm. Second, while absolute light intensity directly affects resolution in intensity-based methods, the NeCam is not constrained in this way, as it relies on relative intensity gradients. This allows the NeCam to mitigate the influence of certain background light levels, thereby improving the signal-to-noise ratio, and to respond to beam probes with even lower light intensities (e.g., 2.5 *µW*, as used for the probe beam intensity in the main text). However, considerations regarding noise tolerance become important under low light conditions—a topic that will be discussed in Supplementary Note 3.


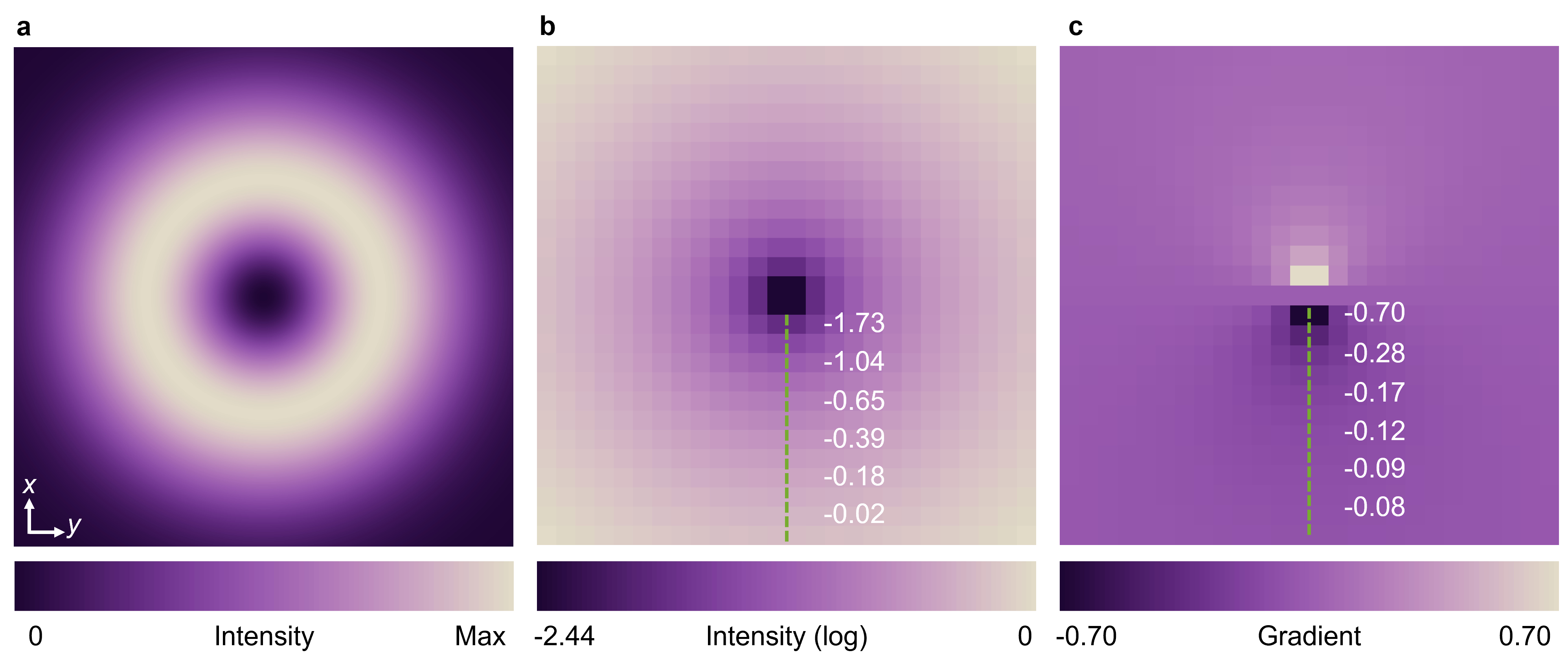


**Fig. S2:**  **Localization limits for singularities using gradient-based and intensity-based methods. a** LG_01_ beam carrying first-order vortex. **b** Logarithmic intensity around the singularity under normalized intensity, where the value indicates the missing Δ*C*_ab_​ required for an intensity response in the corresponding pixel. **c** Logarithmic gradient around the singularity; pixels with absolute values exceeding the NeCam's threshold indicate active event responses. The pixel size in the figure is 4.86 *µm*.

**Supplementary Note 3 – Trailing artifacts of NeCam.**

Trailing artifacts are common phenomena in array-based photodetectors. In NeCam, this effect is light-intensity dependent and primarily occurs in low-light environments^5^, exhibiting characteristics consistent with a resistance-capacitance low-pass filter model^6^. While this potential factor could affect the performance of OSinP, our experiments have not reached the hardware's operational limits. Fig. S3 presents event responses at different rotation velocities, with data originating from Fig. 4 in the main text. As rotation velocity increases, the polarity event responses from singularities gradually grow until reaching saturation, with notable trailing artifacts observed in positive polarity events. These trailing events may become aliased with genuine signals during rotation sensing, potentially affecting the signal-to-noise ratio. In our main experiments, the singularity localization and angle calculation methods overcome this effect, while light intensity adjustment also provides some mitigation. As seen in Fig. S3c, the trailing artifacts do not scale proportionally with rotation velocity but rather result from combined factors including illumination conditions, hardware response delay, and data transmission speed. In principle, the topological charge map of composite vortex beams also influences trailing artifacts, as the topological charge affects both singularity size/spacing and Doppler shift magnitude.


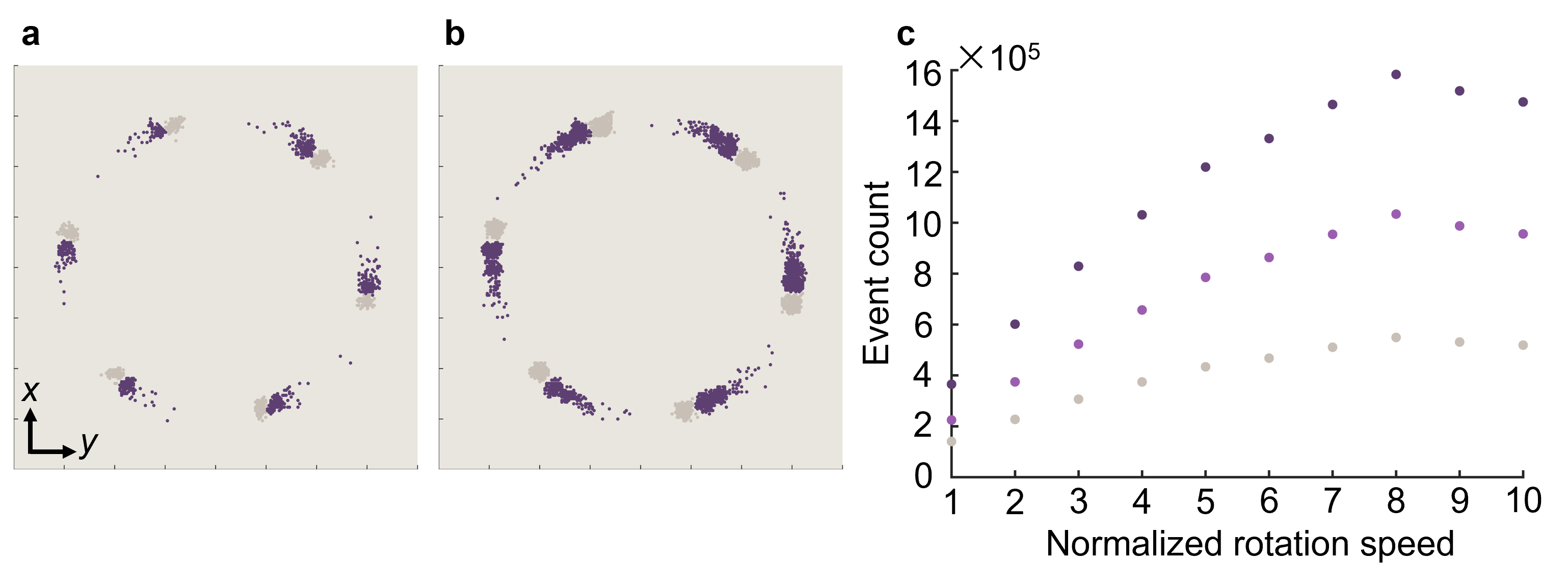


**Fig. S3:** **Trailing artifacts in NeCam responses. a** Event polarity responses at low rotation velocity. **b** Event polarity responses at high rotation velocity, showing trailing artifacts of positive polarity events. **c** Increased event responses with rising rotation speed, where the proportion of trailing positive-polarity events grows until reaching saturation.


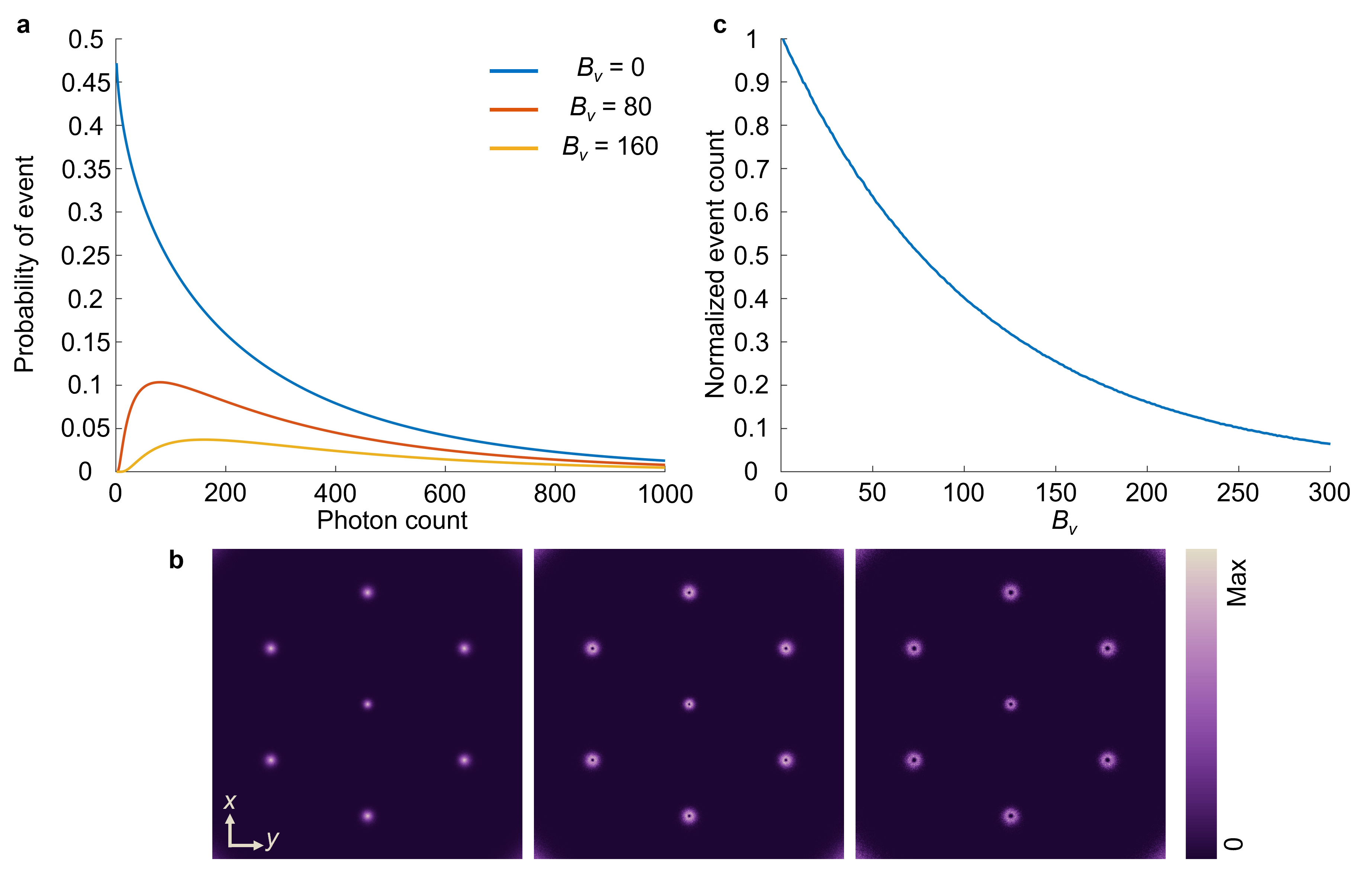


**Fig. S4:** **Noise tolerance by tunable bias in the NeCam for null intensity. a** Theoretical noise event probability as a function of the average photon count, with different curves representing different bias (*B*_v_). **b** Simulated photon noise signals in the structured singularity probe, with bias values of 0, 80, and 160 from left to right, consistent with the conditions shown in **a**. **c** Simulated noise event count as a function of *B*_v_, demonstrating the critical role of *B*_v_ in achieving noise tolerance.

In addition to the trailing effect generated during motion, noise events arising in static conditions can be modeled^7^. In a static scenario, considering photon noise, the event generation process can be expressed in the form of a probability density function:

$P(\lambda)=\frac{1}{2}(1-erf[\frac{(\lambda+B_{v})(e^{C}-1)}{\sqrt{2\lambda(1+e^{2C})}}])$ (S11)

where *λ* is the average photon count, *C* is the threshold and *erf* denotes the error function. This model accounts for the effect of the tunable bias voltage in the event camera, denoted as *B*_v_. In static conditions, an ideal scenario with no intensity variation should produce no events. however, Fig. S4a shows how this probability density function varies with the average photon count when *C* = 0.1, illustrating that at low average photon levels, the probability of events generated by photon noise is significant. Since our focus is on singularities with null intensity, this noise effect is unavoidable. Therefore, considering bias modulation is absolutely essential. Fig. S4a demonstrates how *B*_v_ suppresses noise under low-intensity conditions, and the corresponding noise simulation in OSinP is shown in Fig. S4b. In the structured singularity probe, non-singularity regions possess sufficient intensity to overcome photon noise, while at singularity points, noise can also be suppressed by adjusting *B*_v_. Fig. S4c shows that photon noise events decrease as the bias is adjusted—a conclusion that holds for both positive and negative events. Apart from bias voltage, the NeCam includes other tunable hardware parameters that help maintain noise tolerance, although these are not included in this noise model. For generality, we have listed all the specific bias parameters used in this work: bias_diff = 0, bias_diff_off = 77, bias_diff_on = 34, bias_fo = -19, bias_hpf = 0, bias_refr = 101 (for parameter descriptions refer to the website: https://docs.prophesee.ai/stable/hw/manuals/biases.html).

**Supplementary Note 4 – Detailed experimental setup schematic of OSinP.**

The detailed schematic of the experimental setup for OSinP is provided in this section. A laser beam was collimated using a pinhole and a lens. An iris was used to control the illumination area on the DMD, which then shaped the collimated beam to generate composite vortex beams. On the detection side, a 4f imaging system (Abbe imaging) was employed to project the beams onto the NeCam.


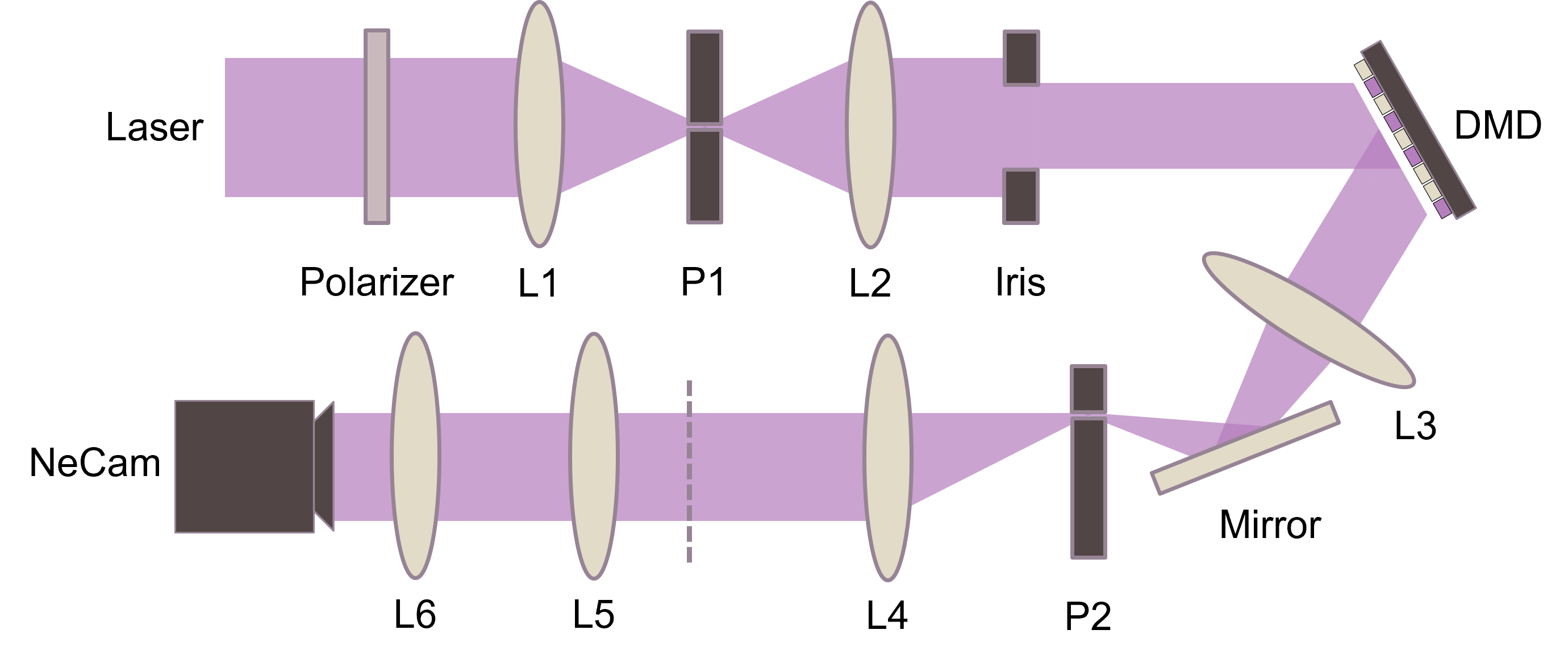


**Fig. S5:** **Detailed experimental setup schematic of OSinP.** L1-6: lens. P1, P2: pinholes.

**Supplementary References**

1. Courtial, J. et al. Measurement of the rotational frequency shift imparted to a rotating light beam possessing orbital angular momentum. *Physical Review Letters* **80**, 3217-3219 (1998).

2. Courtial, J. et al. Rotational frequency shift of a light beam. *Physical Review Letters* **81**, 4828-4830 (1998).

3. Fang, L., Padgett, M. J. & Wang, J. Sharing a common origin between the rotational and linear Doppler effects. *Laser &* *Photonics* *Reviews* **11**, 1700183 (2017).

4. Tang, A. et al. Completely revealing the amplitude properties of Laguerre-Gaussian vortex beams. *Optics Express* **30**, 28892–28904 (2022).

5. Zhang, Z. R. et al. From Sim-to-real: toward general event-based low-light frame interpolation with per-scene optimization. SIGGRAPH Asia 2024 Conference Papers. Tokyo: Association for Computing Machinery, 2024, 99.

6. Liu, H. Y. et al. Seeing motion at nighttime with an event camera. 2024 IEEE/CVF Conference on Computer Vision and Pattern Recognition. Seattle: IEEE, 2024.

7. Cao, R. M. et al. Noise2Image: noise-enabled static scene recovery for event cameras. *Optica* **12**, 46–55 (2025).
